# Microbial Patterns Signaling via Toll-Like Receptors 2 and 5 Contribute to Epithelial Repair, Growth and Survival

**DOI:** 10.1371/journal.pone.0001393

**Published:** 2008-01-02

**Authors:** Renat Shaykhiev, Jürgen Behr, Robert Bals

**Affiliations:** 1 Department of Internal Medicine, Division for Pulmonary Diseases, Philipps-Universtät Marburg, Marburg, Germany; 2 Division for Pulmonary Diseases, Department of Internal Medicine I, Ludwig-Maximilians-Universtät München, München, Germany; New York University School of Medicine, United States of America

## Abstract

Epithelial cells (ECs) continuously interact with microorganisms and detect their presence via different pattern-recognition receptors (PRRs) including Toll-like receptors (TLRs). Ligation of epithelial TLRs by pathogens is usually associated with the induction of pro-inflammatory mediators and antimicrobial factors. In this study, using human airway ECs as a model, we found that detection of microbial patterns via epithelial TLRs directly regulates tissue homeostasis. *Staphylococcus aureus* (*S. aureus*) and microbial patterns signaling via TLR2 and TLR5 induce a set of non-immune epithelial responses including cell migration, wound repair, proliferation, and survival of primary and cancerous ECs. Using small interfering RNA (siRNA) gene targeting, receptor-tyrosine kinase microarray and inhibition studies, we determined that TLR and the epidermal growth factor receptor (EGFR) mediate the stimulating effect of microbial patterns on epithelial repair. Microbial patterns signaling via Toll-like receptors 2 and 5 contribute to epithelial repair, growth and survival. This effect is independent of hematopoietic and other cells as well as inflammatory cytokines suggesting that epithelia are able to regulate their integrity in an autonomous non-inflammatory manner by sensing microbes directly via TLRs.

## Introduction

Epithelial cells (ECs) cover the body surfaces and represent a primary site of host-microbe interactions [1]. As first line of defense, epithelial barriers are responsible for sequestration of microorganisms and their inactivation playing thereby a critical role in the prevention of immune cell activation and infection under steady state conditions [2]. Remarkably, ECs possess an intrinsic capacity to survive despite the continuous exposure to considerable amounts of microorganisms present in the environment which are potentially toxic to host cells, suggesting that some homeostatic forces link antimicrobial strategies to mechanisms that control tissue integrity [3], [4].

Recent evidence supports the role of microbial factors in maintaining the structural integrity of epithelial tissues. It has been shown that MyD88-mediated signaling induced by commensal flora in intestinal mucosal cells is important for the regulation of epithelial homeostasis under steady-state conditions and for the expression of protective molecules in ECs following mucosal injury [5]. The observed effect of bacterial factors on epithelial integrity has been associated with their recognition by innate immune cells of hematopoietic origin that abundantly express PRRs including TLRs [6], [7]. Indeed, it has been found that during mucosal injury macrophages provide signals necessary for the regeneration of the damaged epithelium via modulation of epithelial progenitors [8], [9]. Accordingly, activation of NF-kB, a transcription factor involved in the response to many danger factors including TLR ligands [10], is currently associated with an altered epithelial homeostasis and cancer development [11]. However, until now, it remaines obscure whether microbes are able to induce epithelial repair directly, i.e. independently of inflammatory cells and mediators.

A number of EC-derived molecules, such as trefoil factors [12] or members of the epidermal growth factor family [13], are involved in rapid and autonomous epithelial repair in response to damage. It is also well established that ECs express functional PRRs including TLRs enabling them to respond to microbes by producing a broad spectrum of host defense molecules [14]. Since invasion and colonization of epithelial surfaces by pathogenic bacteria is usually associated with damage to epithelial barriers [15], [16], we hypothesized that detection of microbial patterns by ECs may directly induce a homeostatic repair program necessary for the maintenance of epithelial integrity. In this study, we have found that *S. aureus* as well as various microbial products signaling via TLR2 and TLR5 directly induce epithelial repair, survival and growth, and that such compensatory epithelial responses are mediated by an autonomous non-inflammatory pathway linking TLR and EGFR in ECs. This mechanism is likely involved in the pathogenesis of diseases that are associated with a breach of mucosal barrier function such as asthma, chronic obstructive lung disease, and lung cancer.

## Results

### *S. aureus* increases epithelial repair

**Figure 1:**
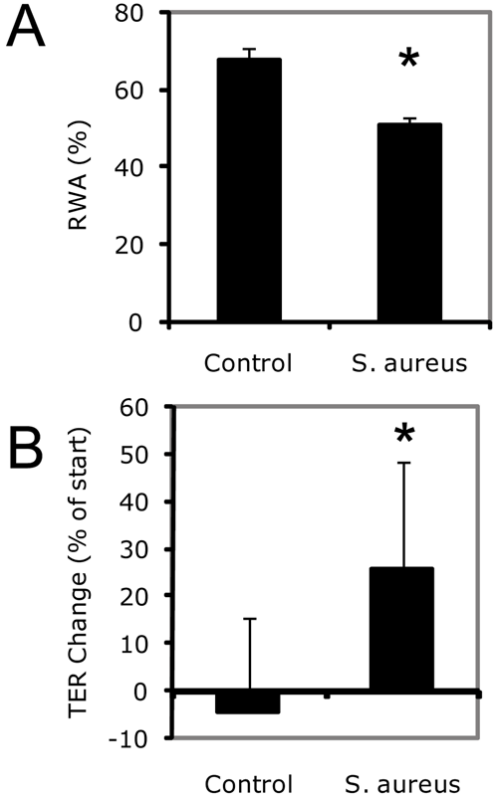
Inactivated *S. aureus* stimulates epithelial wound repair. Mechanically wounded NCI-H292 cell monolayers (A) and differentiated human PBECs (B) were exposed to heat-inactivated *S. aureus* (∼0.5×10^8^ CFU/ml) or medium alone (control). Wound repair was assessed by measuring the remaining wound area (RWA) for NCI-H292 cells (n = 5 in both groups; * P<0.001) and TER for PBECs (n = 6 in control group; n = 17 in *S. aureus* group; * P<0.01) 24 h after injury. Bars represent SD.

To test whether bacteria are able to induce epithelial repair, we selected an experimental *in vitro* model, in which wounded airway epithelium is stimulated with inactivated whole *S. aureus*, one of the major human pathogens able to colonize mucosal surfaces and to extensively interact with ECs of barrier organs [17]. We applied heat-killed *S. aureus* to wounded monolayers of NCI-H292 human airway ECs and found substantially accelerated wound closure (Fig. 1A). To determine whether *S. aureus* exerts a similar effect in polarized primary epithelium, inactivated bacteria were applied to mechanically injured primary human bronchial ECs (PBECs). Repair in this model was assessed by measurement of transepithelial resistance (TER), a physical parameter of tight junctions' integrity that depends on restitution of the epithelial defect and re-establishment of cell-cell contacts [18]. Inactivated *S. aureus* stimulated epithelial repair (Fig. 1B) as determined by significant increase of TER.

### TLR ligands regulate epithelial repair

**Figure 2:**
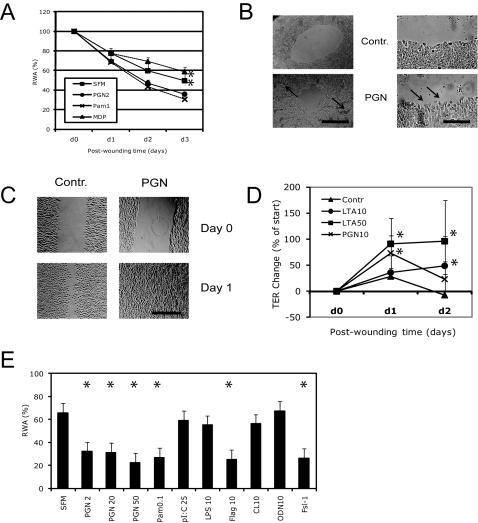
TLR ligands regulate epithelial wound repair and migration. (A) Mechanically wounded NCI-H292 cell monolayers were stimulated with 2 µg/ml PGN, 1 µg/ml Pam_3_CSK4, 10 µg/ml MDP, or SFM alone. RWA was measured during 3 days after damage (n = 3; * P<0.05 as compared to SFM); (B) Representative micrographs of wounds in SFM and PGN groups after 24 h, the areas of increased cellularity (left) and lamellipodia formation (right) are marked with the arrows; bar = 500 µm (left panel);  = 150 µm (right panel). (C) Small linear (∼0.2 mm in width) wounds were generated by scraping of NCI-H292 cell monolayers and cell migration in control and stimulated (10 µg/ml PGN) was analyzed 1 day after injury as shown in the representative micrograph; bar = 300 µm. (D) Differentiated human PBECs were mechanically injured and then stimulated with LTA (10 and 50 µg/ml); TER was monitored during 3 days after wounding (n = 6; * P<0.02); (E) Mechanically wounded NCI-H292 cell monolayers were stimulated with PGN, Pam_3_CSK4 (Pam), poly(I:C), ultra pure LPS, flagellin (Flag), CL097 (CL), and ODN2006 type B at indicated concentrations (µg/ml), or SFM alone; RWA at 24 h after injury was measured (n = 5; * P<0.01). Bars represent SD.

Bacteria display various microbial patterns that can potentially be recognized by host cells. The major cell wall-associated factors expressed by *S. aureus*–PGN and LTA–are well-established ligands for TLR2 [19]. Inside the cell, PGN-derived products can be recognized by the proteins of the NOD family [20]. Thus, we asked whether the observed effect of *S. aureus* on epithelial repair is a result of detection of microbial patterns by ECs. To test this, we stimulated wounded NCI-H292 epithelial monolayers with individual microbial factors and found that PGN significantly increased wound repair (Fig. 2A and 2B). Notably, PGN induced the formation of areas with increased cell density around the wounds (Fig. 2B) indicating that EC proliferation is stimulated simultaneously with induction of the wound closure process. Analysis of wound edges revealed an increased presence of lamellipodia formation in PGN-stimulated cells (Fig. 2B) suggesting that recognition of the microbial pattern promotes EC migration. Also in very small epithelial wounds (∼0.2 mm in width), whose closure is dependent exclusively on rapid cell migration, addition of PGN resulted in a complete coverage of epithelial defects (Fig. 2C), whereas in the control group a considerable wound area remained uncovered. There was no difference between PGNs isolated from *S. aureus* and *B. subtilis* in terms of their impact on epithelial repair (data not shown). Synthetic TLR2 ligands such as Pam_3_CSK4 (Fig. 2A), FSL-1 (Fig. 2E), and MALP-2 (data not shown) also accelerated wound closure. In contrast, the NOD2 ligand MDP did not stimulate epithelial repair (Fig. 2A). Application of LTA (Fig. 2D) and PGN (data not shown) to injured differentiated PBECs resulted in a more rapid TER recovery. Notably, TLR5 ligand flagellin also increased epithelial wound closure (Fig. 2E), suggesting that the observed phenomenon is not restricted to patterns signaling via TLR2. While traditional not re-repurificated LPS preparation effectively stimulated epithelial repair (Fig. S1), the ultra-pure LPS induced only a very weak effect (Fig. 2E). Interestingly, the TLR3 ligand poly(I:C), TLR7/8 ligand CL097, and TLR9 ligand CpG ODN2006, all known to interact with their receptors within the endosomal compartment, did not stimulate but rather delayed epithelial repair (Fig. 2E).

### TLR agonists modulate epithelial growth and survival

**Figure 3:**
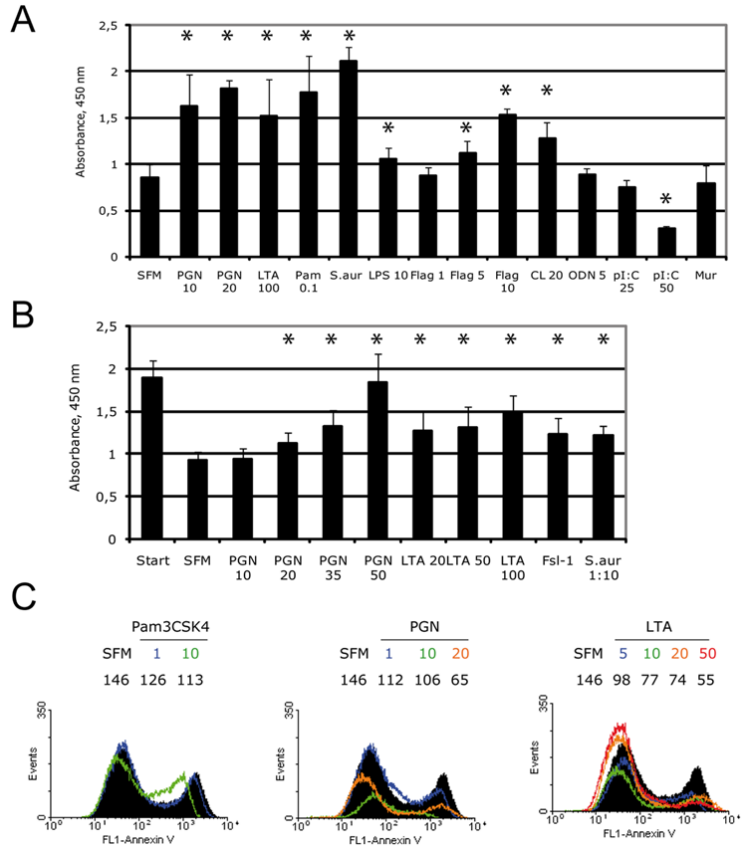
TLR ligands induce epithelial growth responses and protect from apoptosis. (A) Subconfluent NCI-H292 cells or (B) human PBECs were stimulated with selected TLR or NOD ligands including PGN, LTA, Pam_3_CSK4 (Pam), LPS, flagellin (Flag), CL097 (CL), poly(I:C) (pI:C), ODN2006 type B (ODN), murabutide at indicated concentrations (µg/ml), heat-inactivated *S. aureus* (∼0.5×10^8 ^CFU/ml), or SFM alone; after 24 h the numbers of metabolically active cells were measured by the WST assay (n = 8, * P<0.05 as compared to SFM). NCI-H292 cells were assayed after 18 h of serum starvation, PBECs-without serum starvation. In B, the value of starting WST measurement at the beginning of experiment (before changing to serum-free conditions) is shown as “Start”. (C) Semi-confluent human PBECs were exposed to TLR2 ligands Pam_3_CSK4, PGN, or LTA for 24 h. The numbers of apoptotic cells, which correlate positively with binding to exogenously added annexin V, were analyzed by an annexin-binding assay using FACS. Black histograms refer to SFM group, and colored histograms refer to different stimuli (concentrations are indicated at the upper rows immediately below the names of the groups). At the lower rows, the mean fluorescence intensity (MFI) of annexin binding is indicated. The data of representative experiments is shown. Bars represent SD.

Interaction of microbes with ECs occurs continuously, not only during epithelial injury, and may play a role in maintaining epithelial barrier integrity [21], [22]. To test whether microbial factors contribute to epithelial growth under steady-state conditions, we first used the WST assay that allows quantification of metabolically active, viable cells in culture. We found that heat-killed *S. aureus*, PGN, LTA, Pam_3_CSK4, flagellin (Fig. 3A) and MALP-2 (data not shown) increased NCI-H292 cell number. In contrast, poly(I:C), CpG, and the NOD ligand murabutide did not stimulate EC proliferation (Fig. 3A). Whereas not re-purified LPS strongly increased EC numbers (Fig. S1), ultra pure LPS exerted significant but relatively weak effect on EC proliferation (Fig. 3A). The BrdU assay, confirmed the mitogenic effect of PGN, Pam_3_CSK4, and flagellin on NCI-H292; however, the TLR3 ligand poly(I:C) decreased BrdU incorporation (Table 1).

**Table 1:** TLR ligands regulate epithelial proliferation.

	NCI-H292		PBECs	
Group	Mean±SD	P	Mean±SD	P
SFM	0.126±0.009		0.091±0.005	
PGN2	0.134±0.005	<0.05	0.095±0.009	<0.05
PGN10	0.140±0.007	0.002	0.094±0.007	NS
PGN50	0.148±0.006	<0.001	0.098±0.008	<0.05
Pam10	0.143±0.007	<0.001		
LPS50	0.140±0.005	0.001		
Flag10	0.140±0.008	<0.01		
CLO20	0.125±0.006	NS		
PolyI:C25	0.119±0.006	<0.05		

Subconfluent NCI-H292 cells and PBECs were stimulated with different TLR agonists at indicated concentrations (µg/ml) or SFM for 24 h; thereafter cell proliferation was analyzed by a BrdU ELISA-based assay, as described in Methods; the absorbance data shown here directly corresponds to the BrdU incorporation by the cells.

Next, we examined whether other epithelial cancer cell lines respond to TLR signals by altered proliferation. Indeed, addition of PGN to the non-small cell lung carcinoma cells U1810 and to the cervical adenocarcinoma HeLa cells induced statistically significant increase of cell number in a WST assay (Table 2). LPS and flagellin also increased HeLa cell number (Table 2). Since increased proliferation after exposure to the TLR ligands may be a feature of cancerous cells, we examined the effects of microbial factors on survival and proliferation of PBECs grown as submersed nondifferentiated monolayers. As shown in Figure 3C, serum starvation during 24 h resulted in a considerable decrease of primary EC number. However, addition of TLR2 stimuli significantly increased the number of viable cells 24 h after stimulation as compared to the control group. Inactivated *S. aureus* and FSL-1 showed a similar protective effect (Fig. 3B). To support these data, we performed a BrdU assay and found that PGN increased proliferation of PBECs in a dose-dependent manner (Table 1).

**Table 2:** TLR ligands regulate proliferation of cancer cells.

Cells	Group	Mean±SD	P
**U1810**	SFM	1.1±0.1	
	PGN50	1.3±0.15	<0.03
**HeLa**	SFM	1.235±0.04	
	PGN20	1.32±0.03	<0.001
	PGN50	1.36±0.03	<0.001
	LPS50	1.31±0.05	0.005
	Flag10	1.41±0.05	<0.001

Subconfluent U1810 and HeLa cells were stimulated with indicated TLR ligands at indicated concentrations (µg/ml) or SFM for 24 h; thereafter numbers of metabolically active cells were measured by a WST proliferation assay; the absorbance data directly corresponds to the numbers of viable cells; n = 8, * P values indicated as compared to SFM.

To determine whether TLR agonists can protect ECs from apoptosis, we tested the ability of the TLR2 ligands PGN, LTA, and Pam_3_CSK4 to rescue PBECs from death caused by 1 day of serum starvation. Indeed, application of these agonists resulted in a substantial decrease in numbers of apoptotic cells (Fig. 3C).

### Microbial products induce epithelial repair by activating a TLR–EGFR pathway

**Figure 4:**
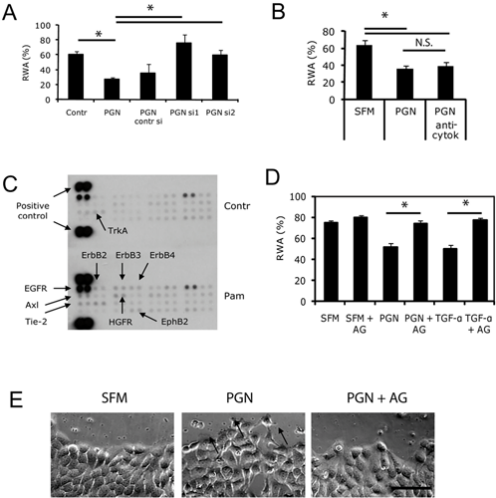
Microbial factors regulate epithelial repair using TLR and EGFR pathways independently of inflammatory cytokines. (A) At the day of seeding, NCI-H292 cells were transfected with 5 nM of one of two different siRNAs against TLR2 (si1 and si2), control siRNA (contr si), or remained untransfected; 48 h after, the cells were subjected to serum-starvation for 16 h. Confluent monolayers were wounded mechanically and then immediately stimulated with 10 µg/ml PGN or SFM; 24 h after stimulation the RWA was measured (n = 5; * P<0.001 as compared to the PGN group, N.S. = not significant). (B) Mechanically wounded NCI-H292 cell monolayers were stimulated with 10 µg/ml PGN in the presence of a mixture of neutralizing monoclonal antibodies against cytokines IL-1β, TNF-α, IL-6, IL-8 (each at 1 µg/ml); RWAs were calculated at the end of day 1 after injury (n = 4 in PGN group, n = 5 in other groups; * P<0.001, N.S. = not significant). (C) Serum-starved NCI-H292 cells were stimulated with 1 µg/ml Pam_3_CSK4 (Pam) or SFM (control) during 10 min and then lysed with RIPA, centrifuged and subjected for phospho-receptor tyrosine kinase (RTK) antibody microarray; representative membranes corresponding to control (upper) and Pam (lower)-stimulated cells are shown; activated RTKs are indicated. (D-E) Confluent NCI-H292 monolayers were exposed to the selective inhibitor of EGFR tyrosine kinase AG1478 (AG, 1 µM), 1 h before and immediately after wounding prior to addition of 10 µg/ml PGN, 20 ng/ml TGF-α, or SFM, or treated with same stimuli without exposure to AG1478; (D) RWAs were measured 24 h after wounding (n = 5, * P<0.001). (E) Wound edges of NCI-H292 monolayers were analyzed for cell migration; micrographs of representative experiment; asterisk = lamellipodia formation; bar = 500 µm. Bars represent SD.

Various families of PRRs are involved in the recognition of microorganisms [23] and bacteria including *S. aureus* can interact with more than one receptor on the host cell simultaneously [24]. Therefore, it was important to determine the specific PRRs that link innate immune recognition to epithelial growth and repair. The ability of flagellin, known to signal solely via TLR5 [25], to induce epithelial wound closure and proliferation, directly indicated the involvement of the TLR (Fig. 2E and 3A). However, this was not clear for PGN, which is able to interact with both TLR2 and NOD [26]. In order to confirm the involvement of TLR2, we transfected NCI-H292 cells with siRNA against TLR2 and found that such inhibition resulted in abrogation of effect of PGN on epithelial wound healing (Fig. 4A). In favor of the involvement of TLR2, the specific TLR2 agonists Pam_3_CSK4 (Fig. 2E) and FSL-1 increased both wound repair (Fig. 2E) and EC proliferation (Fig. 3A and 3B), whereas the NOD ligands MDP (Fig. 2A) and murabutide (Fig. 3A) were not effective to induce these effects.

Another question was whether TLR signals modulate epithelial homeostasis directly or due to the effect of secondary mediators released from ECs stimulated via TLRs. Indeed, we found that microbial factors that accelerated wound closure induced release of IL-1β, TNF-α, IL-6 and IL-8 (Fig. S2). These cytokines are known to play a role in stimulating migration and/or proliferation of cancerous ECs [27]. However, even highest concentrations of these cytokines detected in cell supernatants (IL-8-3 ng/ml, IL-6-1 ng/ml, TNF-α–0.3 ng/ml, and IL-1 β–0.1 ng/ml) were not sufficient to accelerate wound closure (data not shown). Independence of TLR-mediated repair from inflammatory cytokines is further supported by the observation that the TLR3 ligand poly(I:C), which exerted detrimental effect on ECs, induced the highest levels of cytokines as compared to other TLR agonists (Fig. S2). Finally, a mixture of blocking monoclonal antibodies (mAbs) against IL-1β, TNF-α, IL-6, and IL-8 had no significant effect on PGN-stimulated wound healing (Fig. 4B).

To gain insight into signaling mechanisms, we stimulated NCI-H292 cells with the TLR2 agonist Pam_3_CSK4 and analyzed the phosphorylation of 42 different receptor tyrosine kinases (RTKs) using a human phospho-RTK microarray. We detected that activation of TLR2 in ECs induced phosphorylation of a set of RTKs implicated in epithelial repair, growth and carcinogenesis (Fig. 4C). Notably, EGFR (ErbB-1) and all other members of EGFR family HER2/c-neu (ErbB-2), Her 3 (ErbB-3) and Her 4 (ErbB-4) were among the most activated RTKs. In addition, Axl, EphB2, HGFR, Tie-2, and TrkA were also activated (Fig. 4C). Based on the microarray data indicating the link between TLR2 and EGFR, we evaluated the relevance of EGFR-mediated signaling for epithelial repair induced by TLR2 signals using the selective EGFR inhibitor AG1478. This substance completely blocked the effect of PGN on the healing of mechanically wounded NCI-H292 cell monolayers (Fig. 4D). Specifically, the cell migration from the wound edges was affected (Fig. 4E), indicating that increased EC migration during wound repair induced by TLR2 signals is an EGFR-mediated process.

## Discussion

ECs form a physical barrier against environmental factors, including microorganisms [28]. Important feature of surface epithelia as barrier tissue is their ability to resist injury which can potentially be induced by every pathogen interacting with ECs. Mucosal pathogens and their products have been traditionally associated with epithelial death, tissue injury and inflammation [29], [30]. The main finding of this study is the ability of microbial patterns to regulate epithelial homeostasis during direct interaction with ECs. We propose a model in which damaged ECs respond to microbes by increased repair. Using airway epithelium as a prototypical barrier tissue, we found that incativated *S. aureus* and various bacterial products signaling via TLR2 and TLR5 can directly stimulate epithelial wound repair and increase proliferation and survival of uninjured ECs. The NOD proteins that recognize microbial patterns in the cytosol and endosomal TLRs do not exert such effects. To the best of our knowledge, this is the first study demonstrating that bacteria as well as microbial patterns signaling via TLR2 and TLR5 directly modulate a whole panel of non-immune responses related to repair and homeostasis in human ECs.

The role of microbial factors in the regulation of structural integrity of mucosal tissues has been investigated in several recent studies. It has been shown that MyD88 signaling controls the balance between EC proliferation and differentiation under steady-state conditions and that recognition of commensal bacteria by mucosal cells is critical for the maintenance of intestinal homeostasis [31]. Further studies revealed that macrophages and stromal cells are involved in epithelial repair by the recognition of invading microbes and production of factors regulating epithelial progenitor cell activity [32], [33]. Our study provides evidence that bacteria and associated factors can directly induce repair-related processes in ECs independently from bone marrow-derived cells and inflammation. In support of our finding, one recent study revealed increased epithelial proliferation in the colon of mice, whose ECs were deficient in the single immunoglobulin IL-1 receptor-related molecule (SIGIRR), a negative regulator for Toll-IL-1R signaling [34]. Our data raise an intriguing possibility that, under physiological conditions, the presence of the small amounts of certain microorganisms may control epithelial barrier integrity without developing inflammation.

An important finding of the present study is the observation that distinct TLRs mediate different effects on epithelial homeostasis. Whereas microbial factors signaling via cell-surface expressed TLR2 and TLR5 increased epithelial wound closure, proliferation and survival, ligands of endosomal TLRs mediated an opposite effect. In agreement with our data, TLR2 and TLR5 signaling have been found to be important for maintaining tight junctions assembly [35] and cytoprotection [36] in intestinal ECs. Our study adds to these observations, providing evidence that interaction of microbial factors signaling via these PRRs with ECs results in a more rapid repair of the injured epithelium and contributes to maintenance of EC turnover under steady-state. Notably, stimulation of endosomal TLRs, including TLR3, TLR7/8, and TLR9, did not induce these effects and even caused death of ECs and delayed epithelial wound closure. Importantly, expression of these receptors in cells analyzed in our study have been previously determined at levels comparable with TLR2 and TLR5 [37] suggesting that the inefficacy of endosomal TLRs in inducing epithelial repair is likely due to their signaling particularities. The toxic effect of such signaling in ECs is likely due to the pro-apoptotic action of type I IFNs normally induced upon stimulation of endosomal TLRs [38]. Accordingly, type I IFNs antagonize proliferation and survival of fibroblasts caused by stimulation of TLR3 and TLR4 [39]. A similar mechanism may operate in ECs. In agreement with our data, rotavirus double-stranded RNA is known to induce TLR3 signaling and to cause apoptosis and delayed repair in rat intestinal ECs [40]. Two recent articles reported the role for TLR4 in epithelial wound closure [41] and viability of intestinal ECs [42]. We did not observed a significant effect on wound repair when ultra pure LPS was used, suggesting that the presence of a small amounts of other microbial products signaling via TLR2 and or TLR5 in not ultra-pure LPS preparations may be a reason for such discrepancy [43]. There are several distinctive features of TLR4 signaling that may explain why ultra pure LPS does not exert EC responses as do TLR2 and TLR5. First, as compared to other non-endosomal TLRs, signaling via TLR4 may involve MyD88-independent pathway responsible for type I IFN production [44], that may negatively influence survival and repair of ECs. Second, expression of TLR4 within intracellular compartments of ECs has been reported [45] and presence of additional factors may be critical for LPS delivery and induction of epithelial responses via TLR4.

Three carcinoma cell lines, bronchial mucoepidermoid carcinoma-derived cells NCI-H292, non-small cell lung carcinoma cells U1810, and cervical adenocarcinoma cells HeLa, responded to TLR2 stimulation by increased proliferation, suggesting that TLR2 ligands could act as direct tumor-promoting factors. Ligands of TLR2 and TLR5 also induced migration of NCI-H292 cells, suggesting that both cancer growth and spreading can be affected by certain TLR signals. It has been previously shown that LPS increases DNA synthesis in colon carcinoma cells [46] and growth of epithelial ovarian cancer cells [47]. In both studies, however, the effects have been suggested to be induced through the generation of inflammatory signaling in ECs. Although, we also detected increased release of pro-inflammatory cytokines from TLR-stimulated airway ECs, the concentrations detected were insufficient to induce the effects on epithelial repair, supporting our idea that the interaction between innate immune recognition and epithelial repair represents a phenomenon distinct from a well-established link between inflammation and cancer [48]. Consistent with our data, higher concentrations of inflammatory cytokines were necessary to induce epithelial migration or proliferation in previous studies [49]–[52].

Looking at the molecular mechanism coupling TLR signaling to epithelial homeostasis, we found that stimulation of epithelial TLRs induces activation of a panel of receptors with tyrosine kinase activity. Notably, all four members of the EGFR family including EGFR (ErbB-1), HER2/c-neu (ErbB-2), Her 3 (ErbB-3), and Her 4 (ErbB-4) displayed the maximal increase of phosphorylation. Although microarray analysis revealed that EGFR is the principal RTK regulated by TLR2 signaling, several other RTKs were also significantly activated following TLR2 engagement. Some of them, like Axl [53], EphB2 [54], HGFR [55], and the nerve growth factor receptor TrkA [56] are implicated in epithelial repair, proliferation, and cancer growth. Our data show that EGFR plays a predominant role in mediating wound healing induced by TLR2 signals in ECs, since specific blockade of the RTK associated with this receptor completely abolished the effect of PGN on epithelial migration and repair. Interestingly, some recent studies also revealed that microbial factors interfere with epithelial EGFR signaling. *S. aureus* and associated factors, LTA, protein A, and α-toxin, have been found to elicit airway mucus production [57], alter TNFR signaling [58] and induce a mitogenic effect [59] in ECs by activation of EGFR. The present study demonstrates that homeostatic cell responses depend on TLR signaling which is coupled to EGFR activation in ECs.

Taken together, we provide evidence for the presence of a direct link between TLR-mediated recognition of microbes by ECs and epithelial homeostasis. TLR signals induce an autonomous compensatory program in ECs resulting in increased migration, proliferation, survival, and wound repair. This mechanism operates independently of inflammatory cells and cytokines, involves activation of EGFR, is likely involved in regulation of epithelial integrity under steady-state conditions as well as during infection, tissue damage, and cancer development, and, therefore, may be of particular scientific and clinical importance.

## Materials and Methods

### Cell culture

Primary human bronchial ECs (PBECs) were isolated from large airways resected during surgery and cultivated either as conventional submersed cultures or as air-liquid interface (ALI) cultures as described [60]. The protocol was approved by the ethics committees of the Universities of Marburg and München, and informed consent was obtained from the donors. Donors (42, 39, 53 years of age) underwent lung transplantation due to pulmonary fibrosis. Results did not differ between cells from the donors. The human EC lines NCI-H292, U1810, and HeLa (ATCC, Manassas, VA) were cultured at 37°C in 5% CO_2_ in RPMI 1640 medium (Gibco, Grand Island, NY) supplemented with 2 mM L-glutamine, 100 U/ml penicillin, 100 µ/ml streptomycin (PAA Laboratories GmbH, Pasching, Austria) and containing 10% heat-inactivated FBS (Gibco). All experiments were performed under serum-free conditions after 16–24 h serum starvation period unless otherwise indicated.

### Wound repair assay and EC stimulation

Epithelial wound closure assay was performed as described [60]. NCI-H292 cells were grown to confluence in 6-well tissue culture plates. Circular (∼1.0–2.0 mm in diameter for conventional wound closure experiments) or linear (∼0.2 mm in width for analysis of rapid cell migration) wounds were scraped in each well with a sterile 10-µl pipette tip. The wounded monolayers were rinsed with serum-free medium (SFM) to remove cellular debris and then different stimuli were applied. The wound area was determined using inverted phase contrast microscope (Axiovert 25; Carl Zeiss GmbH, Oberkochen, Germany), using the Evolution LC Megapixel FireWire Camera Kit bundled with Image-Pro Discovery software (Media Cybernetics, Inc., Silver Spring, MD) immediately after wound creation and at different post-wounding time-points and expressed as “remaining wound area” corresponding to the percentage of the area measured immediately after wounding. To assess the contribution of cell migration to the wound closure process, the wound edges were examined at different time points after wounding for the presence and intensity of lamellipodia formation using a videomicroscopy technique. To study the repair of differentiated airway epithelial cells, we mechanically damaged the ALI cultures by a sterile pipette tip, scraping off a ring of cells (∼3 mm in diameter) without damaging to the filter support. The stimuli were added in both upper and bottom compartments of ALI culture system to provide the access to both apical and basolateral receptors of ECs. Epithelial repair was evaluated by the measurement of transepithelial electrical resistance (TER) at different time points using an epithelial ohmmeter (EVOM, World Precision Instruments, Sarasota, FL). The results were expressed as TER recovery indicating an increase of TER (%) comparing to the immediate post-wounding value.

Wounded ECs were stimulated with heat-inactivated *S. aureus* (∼0.5×10^8^ CFU/ml) or one of following microbial patterns (all from InvivoGen, San Diego, CA, unless otherwise indicated): peptidoglycan (PGN; *Bacillus subtilis* and *S. aureus*); lipoteichoic acid (LTA; *Steptococcus pyogenes*; Sigma Aldrich), fibroblast-stimulating lipopeptide-1 (FSL-1; *Mycoplasma salivarium*), macrophage-activating lipopeptide-2 (MALP-2; *Mycoplasma fermentans*, 1 µg/ml), Pam_3_CSK4, polyinosine-polycytidylic acid (poly(I:C)), LPS (*E. coli* 0111:B4; Sigma-Aldrich or ultra pure preparations from InvivoGen), flagellin (*Salmonella typhimurium*), TLR7/8 ligand CL097, CpG (ODN2006 type B), NOD ligands murabutide and muramyl dipeptide (MDP; 10 µg/ml; Bachem, Heidelberg, Germany). Transforming growth factor alpha (TGF-α; Sigma-Aldrich; 20 ng/ml), a specific ligand for EGFR, was used as a positive control.

### Analysis of epithelial growth and survival

For analysis of EC proliferation, BrdU labeling and detection kit II, colorimetric BrdU ELISA kit as well as WST-1 proliferation and viability assays were used according to manufacturer's instructions (Roche, Mannheim, Germany). The effect of different stimuli on EC apoptosis was determined by the Annexin binding Vybrant apoptosis assay (Invitrogen, Karlsruhe, Germany) according to manufacturer's instructions. Briefly, were washed with PBS and then briefly trypsinized to obtain single cell suspensions. Trypsin was subsequently inactivated by the addition of culture medium containing 10% FCS and the cells were washed with cold PBS. 10 µl of Alexa Fluor 488-conjugated annexin V and 1 µl of 100 µg/ml of the propidium iodide working solution were added to each 100 µl of cell suspension. After incubation at room temperature for 15 min, annexin-binding buffer was added and the stained cells were analyzed by FACS.

### Cytokine ELISA

Cytokine levels in culture supernatants were determined using a commercially available DuoSet ELISA Development kits for IL-6, IL-8, TNF-α, and IL-1β, according to manufacturer's instructions (R&D Systems).

### Analysis of signaling mechanisms

To determine the involvement of TLRs, NCI-H292 cells (0.5–1×10^6^ cells per well of the 6-well plate) were transfected with 5 nM of TLR2 siRNA (Hs_TLR2_1 HP siRNA or Hs_TLR2_2 HP siRNA, QIAGEN GmbH, Hilden, Germany) or control siRNA (QIAGEN) at the same day as cell planting using HiPerFect transfection reagent (QIAGEN) following manufacturer's instructions. When the cells reached confluence, the medium was changed to SFM, and after 18–24 h of serum starvation, a wound closure assay in the presence or absence of PGN (10 µg/ml) was performed. Neutralizing mAbs against IL-1β, TNF-α, IL-6, IL-8 (all at 1 µg/ml; R&D Systems) and a specific inhibitor of EGFR tyrosine kinase (AG1478, 1 µM; Calbiochem) were used in selected experiments. Human phospho-receptor tyrosine kinase (RTK) antibody Proteome Profiler Array (R&D) analysis was performed according to manufacturer's protocol.

### Statistical analysis

Values are displayed as mean±SD. Comparisons between groups were analyzed by t test (two-sided), or ANOVA for experiments with more than two subgroups. Post hoc range tests were performed with the t test (two-sided) with Bonferroni adjustment. Results were considered statistically significant for P<0.05.

## Supporting Information

Figure S1 LPS induces epithelial growth responses. Subconfluent NCI-H292 cells were exposed to 1 µg/ml LPS (Sigma) or 1% FBS (Contr) and numbers of metabolically active cells were measured by adding WST reagent at the indicated time-points (n = 8, * P<0.01 as compared to SFM).
(1.31 MB TIF)

Figure S2 Stimulation of NCI-H292 cells results cytokine release. Levels of IL-1β, IL-6, IL-8, and TNF-α were detected by ELISA in supernatants of NCI-H292 cells stimulated for 24 h with indicated TLR ligands at indicated concentrations (μg/ml) or SFM alone; data of a representative experiment (one of five).
(7.58 MB TIF)
