# Supplementary figures and images for: Microbial Patterns Signaling via Toll-Like Receptors 2 and 5 Contribute to Epithelial Repair, Growth and Survival
Source: PLoS One. 2008 Jan 2;3(1):e1393. doi: 10.1371/journal.pone.0001393 (PMC2148109; doi:10.1371/journal.pone.0001393)

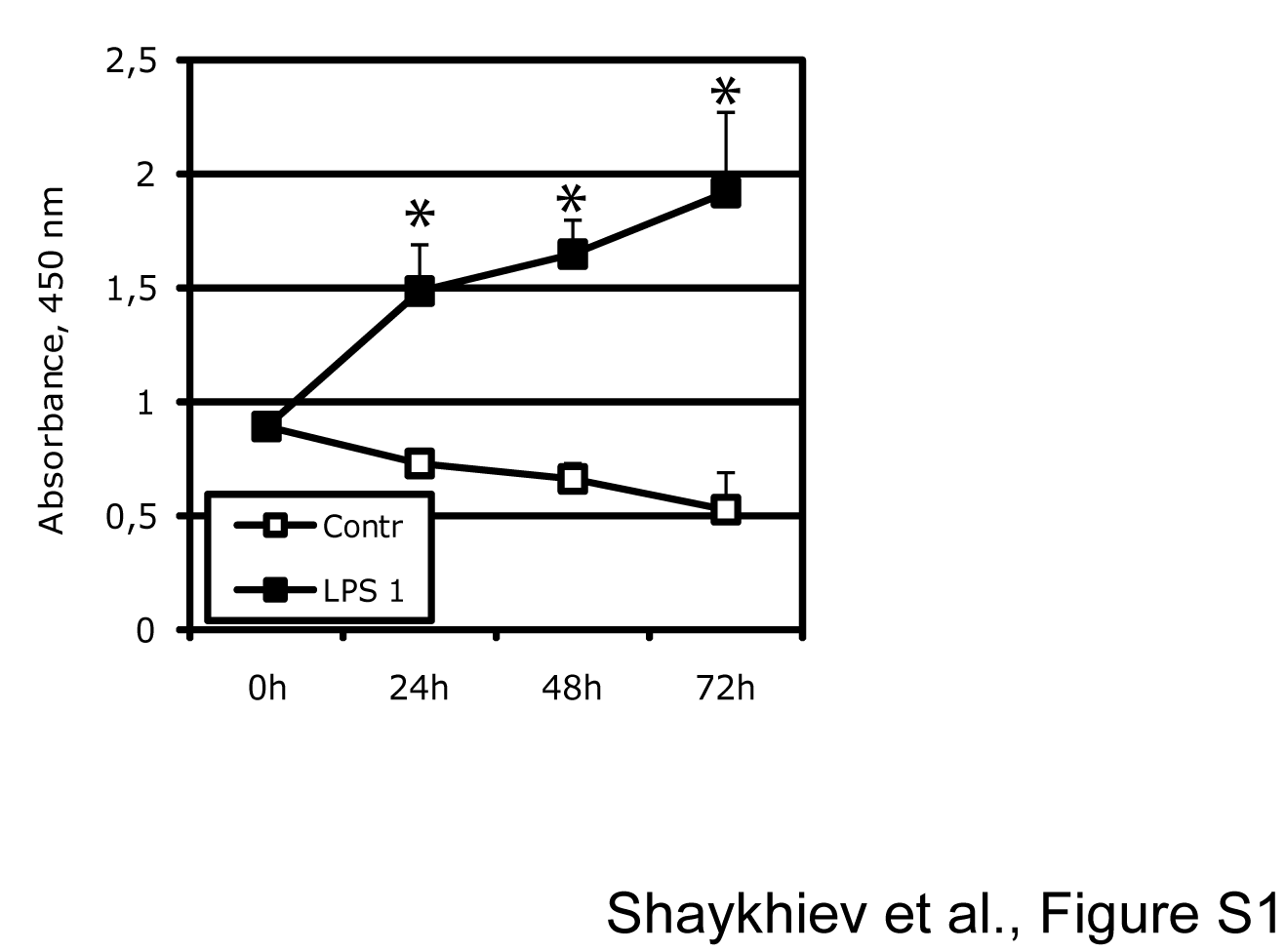

Supplement: Figure S1 — LPS induces epithelial growth responses. Subconfluent NCI-H292 cells were exposed to 1 µg/ml LPS (Sigma) or 1% FBS (Contr) and numbers of metabolically active cells were measured by adding WST reagent at the indicated time-points (n = 8, * P<0.01 as compared to SFM). (1.31 MB TIF) [file pone.0001393.s001.tif]

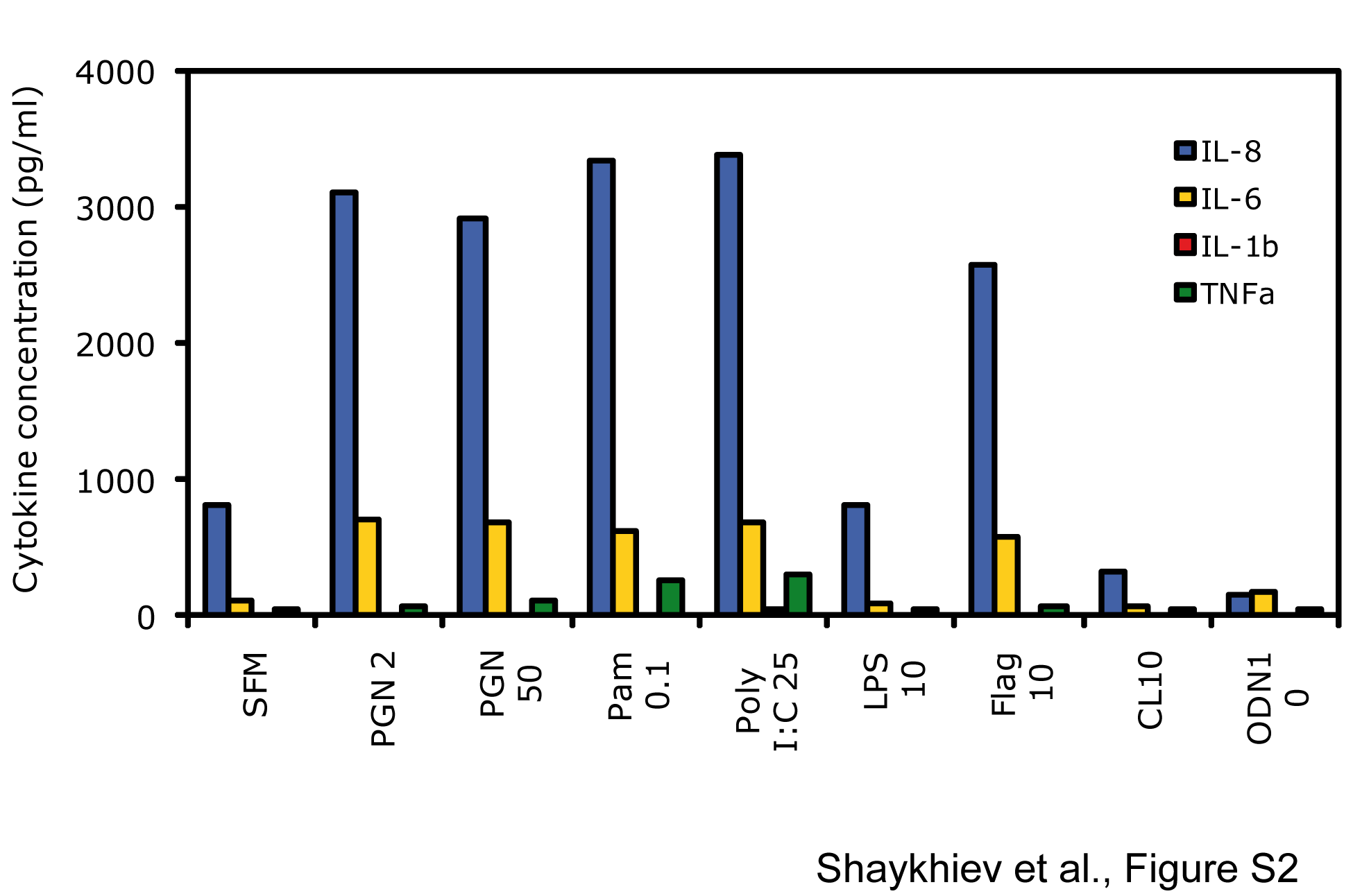

Supplement: Figure S2 — Stimulation of NCI-H292 cells results cytokine release. Levels of IL-1β, IL-6, IL-8, and TNF-α were detected by ELISA in supernatants of NCI-H292 cells stimulated for 24 h with indicated TLR ligands at indicated concentrations (μg/ml) or SFM alone; data of a representative experiment (one of five). (7.58 MB TIF) [file pone.0001393.s002.tif]
